# Giant Right Sphenoid Wing Meningioma as a Reversible Frontal Network Lesion: A Pseudo-bvFTD Case with Venous-Sparing Skull-Base Resection

**DOI:** 10.3390/diagnostics16020224

**Published:** 2026-01-10

**Authors:** Valentin Titus Grigorean, Octavian Munteanu, Felix-Mircea Brehar, Catalina-Ioana Tataru, Matei Serban, Razvan-Adrian Covache-Busuioc, Corneliu Toader, Cosmin Pantu, Alexandru Breazu, Lucian Eva

**Affiliations:** 1 Faculty of General Medicine, “Carol Davila” University of Medicine and Pharmacy, 050474 Bucharest, Romania; 2 Department of General Surgery, “Carol Davila” University of Medicine and Pharmacy, 050474 Bucharest, Romania; 3 Department of Anatomy, “Carol Davila” University of Medicine and Pharmacy, 050474 Bucharest, Romania; 4 Department of Neurosurgery, “Carol Davila” University of Medicine and Pharmacy, 050474 Bucharest, Romania; 5 Clinical Department of Ophthalmology, “Carol Davila” University of Medicine and Pharmacy, 020021 Bucharest, Romania; 6 Department of Ophthalmology, Clinical Hospital for Ophthalmological Emergencies, 010464 Bucharest, Romania; 7 Department of Vascular Neurosurgery, National Institute of Neurology and Neurovascular Diseases, 077160 Bucharest, Romania; 8 Puls Med Association, 051885 Bucharest, Romania; 9 “Nicolae Oblu” Clinical Hospital, 700309 Iasi, Romania; lucianeva@umfro.com

**Keywords:** sphenoid wing meningioma, frontal dysexecutive syndrome, pseudo-frontotemporal dementia, skull-base surgery, venous-sparing resection, neurocognitive recovery

## Abstract

**Background and Clinical Significance**: Giant sphenoid wing meningiomas are generally viewed as skull base masses that compress frontal centers and their respective pathways gradually enough to cause a dysexecutive–apathetic syndrome, which can mimic primary neurodegenerative disease. The aim of this report is to illustrate how bedside phenotyping and multimodal imaging can disclose similar clinical presentations as surgically treatable network lesions. **Case Presentation**: An independent, right-handed older female developed an incremental, two-year decline of her ability to perform executive functions, extreme apathy, lack of instrumental functioning, and a frontal-based gait disturbance, culminating in a first generalized seizure and a newly acquired left-sided upper extremity pyramidal sign. Standardized neuropsychological evaluation revealed a predominant frontal-based dysexecutive profile with intact core language skills, similar to behavioral-variant frontotemporal dementia (bvFTD). MRI demonstrated a large, right fronto-temporo-basal extra-axial tumor attached to the sphenoid wing with homogeneous postcontrast enhancement, significant vasogenic edema within the frontal projection pathways, and a marked midline displacement of structures with an open venous pathway. With the use of a skull-base flattening pterional craniotomy with early devascularization followed by staged internal debulking, arachnoid preserving dissection, and conservative venous preservation, the surgeon accomplished a Simpson Grade I resection. Sequential improvements in the patient’s frontal “re-awakening” were demonstrated through postoperative improvements on standardized stroke, cognitive and functional assessment scales that correlated well with persistent decompression and symmetric ventricles on follow-up images. **Conclusions**: This case illustrates the possibility of a non-dominant sphenoid wing meningioma resulting in a pseudo-degenerative frontal syndrome and its potential for reversal if recognized as a network lesion and treated with tailored, venous-sparing skull-base surgery. Contrast-enhanced imaging and routine frontal testing in atypical “dementia” presentations may aid in identifying additional patients with potentially surgically remediable cases.

## 1. Introduction

Meningiomas are the most common primary intracranial tumors and represent a major source of potential reversible neurological morbidity among older adults. Cognitive and behavioral impairments are becoming increasingly acknowledged as part of the overall clinical burden of meningioma, and are commonly manifest as attentional, working memory, executive function and processing speed issues at the time of surgery [1]. While it is true that mild abnormalities on global screening tests can co-exist with significant clinical declines in function, a small group of patients develop post-surgery persistent or irreversible declines in function. Despite this, the systematic reviews indicate that neurocognitive dysfunction secondary to meningioma has been less consistently defined than in intra-axial tumors, and cognitive outcomes from meningioma treatments have been reported in a variety of underpowered, heterogeneous, and incomplete ways—often utilizing only a few cognitive measures, despite a known and considerable impact on quality of life and independent functioning [2,3]. This suggests that meningiomas should be thought of not only as a structural neurosurgical lesion, but also as a network-level cognitive–behavioral disorder in which both the control of the tumor and the recovery of function should be considered as explicit goals.

Large and frontal meningiomas are particularly relevant because they are more likely to result in executive dysfunction, decreased processing speed and decreased mental flexibility than smaller meningiomas. The preoperative degree of cognitive impairment is associated with the size of the tumor, the presence of vasogenic edema, and the hemisphere affected by the tumor [4].

Furthermore, post-treatment, cognitive and behavioral impairments can persist in a subtle-to-moderate fashion, especially for skull base meningiomas, and many surgical series continue to focus primarily on the evaluation of cranial nerve, visual and motor function and utilize general functional scales rather than specific assessments of frontal-executive or behavioral function [5]. The clinical importance of this deficit in assessing sphenoid wing and medial skull base meningiomas cannot be overstated, since these meningiomas comprise a large portion of meningiomas, anatomically interface with the sphenoid ridge, orbit, cavernous sinus, proximal carotid and middle cerebral arteries, and the anterior visual pathway, and are typically more technically challenging to surgically remove than convexity meningiomas and are associated with a greater risk of cranial nerve injury and lower rates of gross total resection [6].

In spite of these considerations, there are only rare and inconsistent reports of dementia-like dysexecutive, apathetic, or “psychiatric” syndromes related to non-dominant sphenoid wing meningiomas, and there are very few reports describing a giant non-dominant sphenoid wing meningioma causing a behavioral variant frontotemporal dementia (bvFTD)-like syndrome that was supported by quantitative and standardized assessment of both cognitive function and behavior [7].

A current network neuroscience approach provides a useful framework for interpreting how certain extra-axial tumors can produce syndromes that seem to be out of proportion to the anatomical extent of the tumor itself. There is now a growing body of studies using connectomics and resting state fMRI that report changes in connectivity within the DMN, SN and FPN in brain tumors, including meningiomas, that correlate with tumor location, edema and treatment effects [8]. Since these systems are responsible for supporting self-referential processing, goal-directed cognition, and top-down control, they provide a plausible mechanism for the development of dysexecutive–apathetic syndromes seen in large frontal and skull base lesions [9].

Edema and white matter distortion also appear to facilitate the dissemination of the effects of what would otherwise be a seemingly extra-axial lesion through long-range association tracts; thus, providing a mechanistic explanation for how slowly growing meningiomas can generate widespread symptoms of apathy, disinhibition, or dysexecutive syndromes that mimic primary neurodegeneration [10].

Similarly, bvFTD itself continues to be a difficult-to-diagnose entity. Clinical consensus emphasizes the importance of identifying progressive changes in personality, interpersonal conduct, motivation, and executive control in the diagnosis of bvFTD, representing disruption of frontal-insular and anterior cingulate networks. Systematic evaluations have also demonstrated that clinical diagnoses of bvFTD have poor specificity when made outside of specialist centers, and are frequently confounded with psychiatric disorders or atypical structural lesions [11]. This diagnostic ambiguity is further complicated in older adults when neuroimaging is either delayed or restricted to non-contrast studies. Therefore, the overlap between bvFTD and frontal syndromes generated by tumors represents a clinically important zone of ambiguity: patients with slowly progressive apathy, loss of initiative, and executive failure may be directed towards psychiatric or dementia pathways while having potentially reversible mass lesions in the frontal skull base [12].

Given this context, multimodal neuroimaging, along with detailed neurocognitive assessment, offers the potential to recategorize some “degenerative-like” presentations as surgically removable network lesions. Recent research has emphasized the utility of integrating structural MRI, fluid-sensitive sequences, and vascular imaging with domain-specific cognitive batteries in order to improve the prediction of postoperative recovery and to protect critical networks during surgery [13].

However, routine practice still infrequently integrates the following: (i) a bvFTD-level behavioral and cognitive characterization of the patient, (ii) an explicit mapping of edema and mass effect onto frontal-striato-thalamo-cortical and cingulo-callosal circuits, and (iii) a skull-base and venous-sparing microsurgical strategy that is intentionally designed to take into consideration these network factors. The purpose of this case study was to bridge this gap. This case study documents a giant non-dominant sphenoid wing meningioma that produced a completely developed dysexecutive–apathetic syndrome with late generalized seizure activity and pyramidal signs, which were clinically indistinguishable from a bvFTD-like process at initial evaluation. Additionally, the case study documents the patient’s pre- and postoperative neurocognitive, behavioral, and functional scales using granular assessments, and the case study documents the patient’s multimodal imaging, demonstrating how tumor bulk and vasogenic edema distort specific frontal and subcortical networks. Finally, the case study describes how preoperative vascular mapping was utilized to guide a skull-base, venous-preserving pterional surgical approach to decompress these networks, while avoiding ischemic and venous complications. We wish to illustrate three related ideas in this case study through the density of clinical and anatomical correlation. First, that giant frontal and sphenoid wing meningiomas can create pseudo-degenerative syndromes with well-defined dysexecutive–apathetic symptom profiles without hydrocephalus, and that these types of symptom profiles may go undetected unless only global cognitive screens or non-targeted imaging techniques are utilized. Second, that conceptualizing these tumors as network lesions—affecting frontal-striato-thalamo-cortical, cingulo-callosal, and corticospinal pathways—provides insight into the degree of complexity of the clinical presentation and the staged nature of recovery after decompression. Third, that systematic neurocognitive measurements, coupled with meticulous vascular aware skull-base surgical resections, can document and potentially maximize the reversibility of severe frontal syndromes that were initially diagnosed as “psychiatric” or “dementia”.

It is our hope that this case study will provide a practical model for evaluating skull-base meningiomas in the differential diagnosis of atypical frontal dementia presentations and that it will stimulate more comprehensive incorporation of network-based thinking into the neurosurgical management of these seemingly benign appearing tumors.

This study’s contributions are based on three interconnected aspects of the case report:

Clinical Phenotype: The subject exhibited an atypical bvFTD-like dysexecutive-apathy syndrome that was quantitatively measured using neurobehavioral testing methods; however, she had preserved basal motor functions until late in her disease course when she developed generalized seizures as well as evolving pyramidal signs (i.e., pseudodegenerative presentation), which made for a difficult clinical diagnosis prior to brain imaging.

Network Mechanism: Using a network model for interpreting the data derived from multimodal structural and fluid-sensitive MRI studies revealed how the large size of the tumor located in the sphenoid wing outside of the dominant hemisphere caused disruption of several critical networks, including frontal-striato-thalamo-cortical, cingulo-callosal, and corticospinal, due to tumor mass effects and vasogenic edema without hydrocephalus.

Treatment Strategy/Measurable Recovery: A combination of pre-operative vascular mapping (arterial and venous) with a venous sparing approach for a pterional craniotomy with a Simpson-oriented skull base meningioma resection provided a basis for longitudinally measuring the recovery of her cognitive and functional status, demonstrating a “staged” awakening of her frontal network after surgery.

## 2. Case Presentation

The patient is a 70 year old, right handed woman who had been extremely organized and completely self sufficient and had gradually lost all of her executive abilities (as reported by caregiver) due to a 2 year decline: progressing through the loss of ability to perform instrumental functions, requiring assistance with each successive task, until eventually being unable to initiate a task and being mostly non-verbal. The functional assessment indicated that the patient had become completely dependent, although she had maintained some ability to physically move (Lawton-Brody IADL 1/8; mRS 4; Barthel 45/100), indicating a failure of higher-order networks rather than primary motor function. Prior to imaging, quantitative assessments demonstrated a frontal dysexecutive–apathetic syndrome: MoCA 18/30 with significant impairments in trail making, serial 7 s, phonemic fluency (3 words/60 s), and abstraction (0/2) but preserved orientation and repetition; FAB 8/18 with impairments in conceptualization, Luria’s fist-edge-palm sequence, and resistance to interference, indicating damage to both dorsolateral and medial prefrontal networks. Language testing demonstrated dynamic aphasia with impairments in initiating speech and responding to verbal cues (Boston Naming Test 18/30 with improved performance with verbal cues; Token Test 10/12). The memory phenotype was retrieval-based (free recall 0/3 at 5 min with 100% recovery with semantic cues). Neuropsychiatric profiling showed a predominant profile of apathy (NPI-Apathy 18/36) with minimal irritability, indicating a clear dysexecutive–apathetic syndrome.

One day before admission, the patient experienced her first generalized tonic–clonic seizure. Following this episode, the patient’s baseline abulia progressed rapidly to a state of decreased awareness, and the patient exhibited a new weakness in her left side, suggesting acute destabilization of a chronic intracranial process rather than just having one seizure episode. At the time of admission, the patient’s GCS was 11 (E3V3M5); the adapted NIHSS was 9 with decreased level of consciousness (2), difficulty answering questions (2), dysarthria (1), facial weakness on the left (2), and mild weakness in the left arm (1) and leg (1) consistent with a right hemisphere syndrome. During cooperative periods of examination, the patient demonstrated left central facial paresis with activation, increased muscle tone with hyper-reflexia and an extensor plantar response, and MRC 4+/5 strength in the left arm and leg with kinetic coordination deficits. The patient’s pre-admission gait disturbance represented a high risk for falls (Tinneti 14/28) and frailty (Clinical Frailty Scale 6). Systemic/metabolic contributions to her condition were ruled out by laboratory evaluations, including stress hyperglycemia (glucose 8.9 mmol/L) without leukocytosis, mild CRP elevation (3.2 mg/L), and no metabolic derangements, further supporting that there was a primary structural intracranial process leading to quantified frontal collapse, new lateralized findings, and reduced levels of consciousness.

Prior to imaging, a focused, anatomically constrained differential diagnosis was constructed. Giant right fronto-temporal/sphenoid wing meningioma (most likely): the 2 year history of the patient’s frontal syndrome (MoCA 18; FAB 8; NPI-Apathy 18) followed by a late generalized seizure and subsequent progressive lateralized corticobulbar and corticospinal signs, ultimately resulting in the patient’s stupor, provided a syndrome architecture that best fit a slowly expanding extra-axial lesion compressing right frontal-basal ganglia-thalamic-cingulate circuitry. Gradual midline shift probably explained the patient’s reduced level of alertness. Age, sex, and the temporal characteristics of the case supported the likelihood of a meningioma. Right frontal intra-axial glioma: possible; however, the prolonged monotonous course to a late first generalized seizure and the extent of the frontal collapse suggested that there was chronic extrinsic mass effect. Dural metastases: radiographically possible but less likely since there was no identified primary neoplasm, constitutional symptoms, or multiple deficit areas, while future screening would be warranted. Chronic subdural hematomas are also possible in elderly patients, but they would be less consistent with the steady 2-year progression without trauma, the extent of frontal collapse, and late generalized seizure. Behavioral variant frontotemporal dementia: it is a strong clinical mimic; however, the development of later lateralizing seizures, pyramidal signs, and decreased consciousness created a pseudo-bvFTD syndrome architecture, which supports structural pathology over degenerative pathology. Autoimmune/paraneoplastic encephalitis: usually has a subacute onset with psychiatric symptoms, memory loss, multi-focal deficits, limbic seizures, and systemic/serologic features; the unilateral frontal evolution for two years, the absence of multi-focal deficits, and the absence of inflammatory markers make it unlikely. Therefore, prior to imaging, the combination of a quantitatively documented failure of the frontal network (IADL 1/8; mRS 4; FAB 8; NPI-Apathy 18) evolving into a right hemisphere syndrome (NIHSS 9) with reduced consciousness and a first generalized seizure resulted in a convergent syndrome architecture which was specific for a giant right fronto-temporal/sphenoid wing meningioma, thus MRI would represent a directed test of a clearly specified anatomical hypothesis.

**Figure 1:**
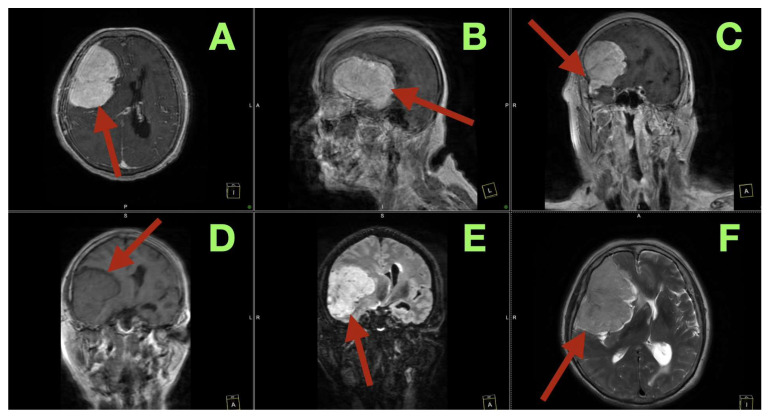
Preoperative MRI of the giant right sphenoid wing meningioma: (**A**) Axial T1 post-contrast image showing a large, homogenously enhancing right fronto-temporo-basal extra-axial mass (arrow) with broad sphenoid wing attachment, CSF cleft, compressed cortical ribbon, slit-like right frontal horn, and leftward midline shift. (**B**) Sagittal T1 post-contrast image demonstrating the full craniocaudal and anteroposterior extent of the tumor over the right frontal convexity, with frontal lobe flattening and bowing of the corpus callosum. (**C**) Coronal T1 post-contrast image highlighting basal dural implantation on the sphenoid wing/fronto-orbital floor and superomedial displacement of the insular–opercular cortex with deformation of the right lateral ventricle. (**D**) Posterior coronal T1 post-contrast image showing medial indentation of the cingulate gyrus and callosal body, consistent with subfalcine displacement. (**E**) Coronal FLAIR image demonstrating extensive vasogenic edema in the right frontal white matter and periventricular zones along frontal projection pathways. (**F**) Axial T2 image depicting the steep tumor–brain interface, broad edema rim, effaced right frontal sulci, and pronounced right-to-left midline shift.

Imaging transformed “unusual dementia with late seizure” into a precisely localized network lesion. Emergency non-contrast CT demonstrated a very large right fronto-temporo-basal extra-axial mass attached to the sphenoid wing that caused effacement of the right frontal sulci, compression of the right frontal horn, and ~1–1.5 cm right-to-left midline shift without hydrocephalus. A high resolution MRI (Figure 1A–F) demonstrated a well-defined, homogenously enhancing extra-axial tumor extending across the right sphenoid wing and right frontal/temporal convexity with a CSF cleft and compression of the cortical ribbon; the falx was displaced medially and the tumor rode over the superior frontal gyrus; the right frontal horn was slit-like and the septum pellucidum deviated leftward (Figure 1A). Sagittal post-contrast images demonstrated near-complete craniocaudal involvement of the right frontal convexity from the orbital roof/sphenoid ridge to the pre-central region, with flattening of the frontal lobe against the inner table and posterior-inferior bowing of the genu and body of the corpus callosum (Figure 1B). Coronal post-contrast images demonstrated broad basal dural attachment along the sphenoid wing and fronto-orbital floor, anterolateral wrapping around the Sylvian fissure, superomedial displacement of the insula and opercula, distortion of the right lateral ventricle with en bloc displacement of the contralateral ventricle and septum, and medial indentation of the cingulate gyrus and callosal body (Figure 1C,D). Fluid-sensitive sequences demonstrated vasogenic edema throughout the right frontal white matter, centrum semiovale, corona radiata, and anterior limb of internal capsule with periventricular extension, tracing fronto-striato-thalamo-cortical and corticospinal pathways and establishing a substratum for the patient’s left pyramidal signs, limb-kinetic dyscoordination, and frontal gait disturbances; the compressed ventricles were not dilated, indicating frontal network failure rather than hydrocephalus (Figure 1E,F).

**Figure 2:**
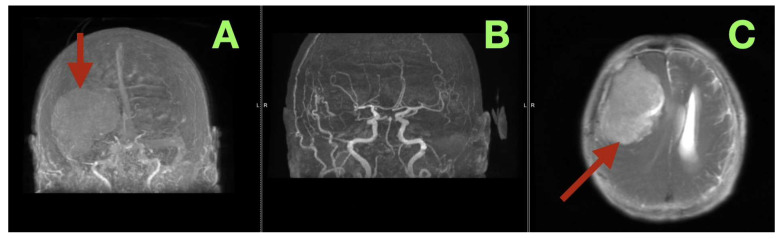
Preoperative vascular imaging and mass effect. (**A**) Coronal contrast-enhanced MR angiographic/venographic reconstruction showing the tumor as a rounded extra-axial filling defect (arrow) in the right anterior–middle cranial fossa, displacing but not occluding cortical veins; superior sagittal sinus and parasagittal collectors remain patent. (**B**) Coronal arterial MRA demonstrating preserved caliber and patency of the right internal carotid and MCA/ACA trunks, laterally displaced but not encased by the tumor. (**C**) Axial T2 image from the vascular protocol illustrating the giant right fronto-temporal extra-axial mass (arrow), surrounding vasogenic edema, severe effacement of right frontal sulci, and right-to-left midline shift, corresponding to the patient’s dysexecutive–apathetic syndrome and left pyramidal signs.

Preoperative vascular studies helped define the boundaries for surgery (Figure 2A–C). Contrast-enhanced MR angiographic/venographic reconstructions (Figure 2A) depicted the tumor as an oval-shaped filling defect in the right anterior-middle cranial fossa, displacing, but not occluding, cortical veins. The superior sagittal sinus and major para-sagittal collectors remained open; superior frontal bridging veins were stretched and draped over the displaced hemisphere; however, they were not invaded. Arterial MRA (Figure 2B) showed that the right internal carotid artery, middle cerebral artery and anterior cerebral artery trunks retained their caliber and remained patent. Lateral displacement, but no encasement or stenosis, was seen in the M2-M3 branches. An intervening arachnoid space existed between the enhancing mass and the Sylvian vasculature, supporting a venous-sparing pterional approach with early debulking and careful arachnoid dissection. An axial T2 slice from the same protocol (Figure 2C) concisely summarized these findings: A giant right fronto-temporal extra-axial mass with a steep interface to the adjacent edematous cortex, severe effacement of the right frontal sulci, and a pronounced right-to-left midline shift, all of which matched the pseudo-bvFTD phenotype with late seizure and focal deficits observed clinically.

An excellent exposure to both the anterior and middle cranial fossae was gained through the pterional craniotomy. The patient was positioned supine, with the head 15 cm above the right atrium, and rotated about 25–30° to the left. This positioning maximized venous drainage and provided the best exposure for the surgeon. A right frontotemporal skin incision was made, and the frontal branch of the facial nerve was identified and protected throughout the interfascial dissection. The temporalis muscle was then elevated subperiosteally to expose the pterion and the frontosphenoidal keyhole. The pterion was then exposed through a right pterional craniotomy.

To improve further exposure and working distance between the tumor and the brain, the skull base was flattened. The lesser sphenoid wing and hyperostotic bone were also drilled to increase the diameter of the corridor to the sphenoid ridge, the orbital roof, and the basal dural attachments. The dura mater was then opened in a curved manner, leaving a cuff of dura mater intact around the superior sagittal sinus and the parasagittal collectors to protect these structures. Due to severe flattening and compression of the right frontal lobe into a thin layer, most of the area contained preserved arachnoid planes, allowing for dissection based on the preservation of arachnoid planes and the protection of the venous system. A compartmentalized, step-wise resection plan was utilized to enable the resection of the tumor in such a way that it would allow the tumor to collapse into a shell before manipulating the Sylvian fissure, the medial veins, and the edematous cortex. The first two steps of this plan involved coagulating the dural feeders to the tumor located along the sphenoid ridge and fronto-basal floor, as well as those located along the orbital roof region. Following the coagulation of the dural feeders, a superolateral capsulotomy was completed, and the initial internal decompression of the tumor was accomplished with suction, bipolar forceps, and ultrasonic aspirator. As the tumor was progressively internally debulked, the venous return through the brain increased, making it safer to circumferentially mobilize the capsule.

Arachnoid-plane dissection was then continued under irrigation using sharp micro-dissection techniques to protect the Sylvian fissure and the cortical veins. The arachnoid overlying the Sylvian cisterns was then sequentially opened to facilitate cerebrospinal fluid egress and further relaxation of the brain. Several M2–M3 vessels were identified as having been displaced by the tumor but not encapsulated by the tumor and were therefore preserved by carefully avoiding the capsular “fulcrums,” gently rolling the capsule away from the vessels using preserved arachnoid planes for visualization and minimal traction. The basal portion of the tumor was then separated from the skull base in small increments and involved opening the cisterns to create slack in the corridor as necessary, while conservatively managing dissection medially at the parasagittal and venous interfaces.

### Venous Interfaces and Simpson Grade of Resection

The parasagittal venous structures and bridging veins were dissected to maintain patency of the veins during the microsurgical dissection process. However, when a clear cleavage plane between the tumor capsule and the vein could not be established without applying traction to the vein or using bipolar forceps on the vein, a thin capsular film was intentionally left attached to the adventitia of the vein as a protective “venous wrapper” to avoid venous thrombosis or infarction. It is important to note that this conservative venous management resulted in no nodular intradural residual mass; a complete examination of the cavity demonstrated no macroscopic tumor, and the attachment to the skull base was completely dissected with focal dural excision/coagulation, and no residual tumor was identified on early postoperative CT imaging. Therefore, the Simpson Grade I designation indicates a gross total removal of the tumor and treatment of the dural attachment, acknowledging that microscopic adherent capsular films may remain at critical venous interfaces to prevent catastrophic venous complications.

After completing the resection of the tumor, a comprehensive assessment of the cavity was conducted to verify whether any residual nodules remained. Hemostasis was achieved using a cortex-sparing hierarchy of techniques, including coagulation of the edges of the dura, topical application of hemostatic agents, and gentle compression with a sponge adjacent to the venous structures, while avoiding direct pial coagulation. The dura was then closed in a watertight fashion, either with a graft and/or onlay reinforcement. The bone flap was replaced with low-profile titanium fixation. An epidural suction drain was placed, and the soft tissue layers were closed in an anatomical sequence.

No intraoperative neurophysiological monitoring was used. Functional and vascular safety was instead achieved by employing strict arachnoid-plane microsurgical dissection techniques, continuous visualization of vessels, and maintaining physiological parameters that emphasized the maintenance of venous outflow.

**Figure 3:**
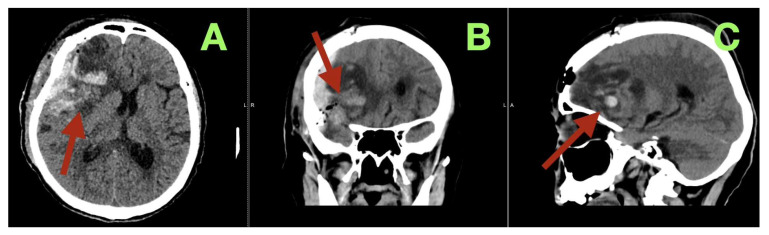
Early postoperative CT (postoperative day 2). (**A**) Axial CT image showing the right fronto-temporo-basal resection cavity occupying the former tumor bed (arrow), with postoperative air and a thin blood rim, reopening of the right frontal horn, near-midline septum pellucidum (residual shift < 3 mm), and absence of residual extra-axial mass or acute hematoma. (**B**) Coronal CT image demonstrating decompression of the right frontal lobe (arrow), reduced mass effect, symmetric or near-symmetric ventricular configuration, and expected postoperative edema without medial frontal venous infarction. (**C**) Sagittal CT reconstruction illustrating the longitudinal extent of the resection cavity along the frontal base and sphenoid wing, with a smooth interface to the remaining brain parenchyma and no radiologic evidence of hydrocephalus or delayed hemorrhage (arrow).

Postoperatively, the patient was managed in the neurosurgical ICU with controlled awakening and narrow physiologic targets (SBP 110–140 mmHg; PaCO_2_ 35–40 mmHg) and close monitoring of sodium (138 mmol/L), osmolality (285 mOsm/kg), and glucose (7.2 mmol/L). The patient awakened slowly, was extubated within 6 h, and had a GCS of 14/15 (E4 V4 M6) with intact brainstem reflexes, symmetric reactive pupils, no new cranial nerve deficits, diminished left pyramidal signs, and no neglect or aphasia; the NIHSS improved from 9 to 5 by POD 1. On POD 2 CT (Figure 3) confirmed complete decompression: a large resection cavity in the right fronto-temporo-basal compartment without residual extra-axial mass, reopening of the right frontal horn, near-midline septum pellucidum (residual shift < 3 mm), decompressed cortical mantle, persistent edema without hemorrhage, medial frontal venous thrombosis, or hydrocephalus; laboratory values remained stable (Na 137 mmol/L; CRP 12.8 mg/L; glucose 6.8 mmol/L), and no early postoperative seizures occurred under levetiracetam prophylaxis.

Cognitive outcomes are only one aspect of neurosurgery’s successful outcomes. No cranial nerve deficiencies resulted from the surgery. Clinically, there were no new complaints related to a decrease in visual acuity or new visual field defects, and the patient had normal ocular motility. The patient did not have symptoms associated with CSF leakage, surgical wound infection, or other infections. Levetiracetam was used for seizure prevention in conjunction with the surgery, and no additional seizures were reported during the patient’s hospital stay or during their follow-up. The patient also did not experience radiological or clinical evidence of venous infarct; delayed hemorrhage; or hydrocephalus secondary to adequate venous drainage and an appropriate degree of brain decompression.

**Figure 4:**
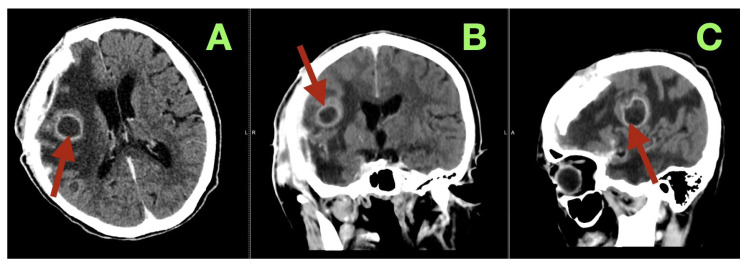
Four-month follow-up CT. (**A**) Axial CT image showing a stable, well-demarcated encephalomalacic cavity in the right frontal region (arrow), with resolved edema, symmetric lateral ventricles, and no nodular extra-axial thickening suggestive of residual or recurrent tumor. (**B**) Coronal CT image illustrating a mature postoperative cavity beneath the right frontal convexity, centered midline structures, and durable normalization of ventricular configuration without venous or hemorrhagic complications (arrow). (**C**) Sagittal CT reconstruction confirming a stable ring-like postoperative cavity along the previous tumor bed, with preserved skull base contours and no new mass effect, consistent with durable decompression and network stabilization (arrow).

Between POD 2 and POD 4, “frontal network awakening” became evident: sustained wakefulness, spontaneous eye contact and conversation without continuous prompting, improved digit span (5 forward/3 backward), and serial subtraction by 3 s, with MoCA improving to 21/30 (executive/attention-focused). FAB improved to 11/18 by POD 4 with better motor sequencing and resistance to distraction; NPI apathy improved from 18/36 to 10/36. Functional recovery progressed rapidly: sitting at bedside with assistance by POD 3, standing and stepping with a walker by POD 4 with reduced magnetic hesitation and improved step initiation; Tinetti improved 14/28 → 18/28 and Barthel 45/100 → 65/100. The epidural drain was removed on POD 3 without CSF leak or wound infection. At discharge (POD 8), the clinical state had diverged markedly from preoperative pseudo-dementia: GCS 15; NIHSS 2 (residual mild facial weakness and left arm drift); mRS 4 → 3; Barthel 75/100; Tinetti 22/28 (supervised safe ambulation); Karnofsky 70%; and CFS 6 → 4. Cognitive consolidation paralleled functional gains (FAB 14/18; MoCA 24/30; Boston Naming Test 25/30; Token Test 11/12; NPI apathy 6/36), consistent with restoration of initiative and motivation. After four weeks of structured neurorehabilitation, at one-month follow-up, she was able to walk unassisted at home and used a cane outdoors; Barthel increased to 85/100, mRS to 2, Karnofsky to 80%, with continuing frontal recovery (FAB 16/18; MoCA 26/30) and no late seizures or systemic complications. Four-month CT follow-up (Figure 4) confirmed durable decompression with a stable right frontal encephalomalacic cavity, resolved edema, symmetric ventricles, and no evidence of recurrence, corresponding clinically to mRS 2, Barthel 90/100, and Karnofsky 90%, with only minor residual executive inefficiency on high-demand tasks.

The quantitative recovery trajectory—NIHSS 9 → 2; GCS 11 → 15; mRS 4 → 2; Barthel 45 → 90; FAB 8 → 16; MoCA 18 → 26—together with ongoing imaging evidence of decompression, demonstrates staged reversal of a severe frontal dysexecutive–apathetic pseudo-bvFTD syndrome. The data demonstrate that the patient’s apparent neurodegenerative phenotype was actually a surgically correctable lesion and that recovery resulted from precise relief of mass effect on frontal and subcortical circuits, rather than nonspecific supportive care. The granular clinical quantification, integrated with multi-modal MRI and vascular mapping, allowed for the conceptualization of the tumor as a reversible network lesion and provided a rationale for a venous-sparing, Simpson-oriented skull base strategy with measurable cognitive and functional recovery.

Since our study does not reflect all patients with pseudo-degenerative syndrome, therefore, it does not provide evidence-based information about how often pseudo-degenerative syndrome is presented clinically, what the typical pattern of postoperative cognitive recovery is, or how each surgery-related factor and each factor related to the perioperative period contribute to the clinical presentation seen in the current case. Moreover, while we provided longitudinal, detailed clinical data with high-resolution images of the brain structure and vascular system, we did not include advanced functional networks (e.g., resting state connectivity, quantitative tractography), which could have provided additional details for the mechanism behind the changes observed. Therefore, the above-described limitations emphasize that the findings described herein can only be viewed as hypothesis-generating. Nevertheless, we believe that the unique contributions of this case are based on the rare integration of three different levels of information including (1) an extremely well-quantified bvFTD-like dysexecutive–apathetic phenotypic presentation with intact fundamental motor skills; (2) a large left-sided sphenoid wing meningioma causing extensive damage and edema to frontal–striatal–thalamic–cortical and cortico-spinal pathways without hydrocephalus; and (3) a sequential and independently verified “awakening” of the frontal network after surgery using a venous sparing, Simpson oriented skull base approach with preoperative vascular mapping. Therefore, we hope that this framework of combined clinical–anatomical–vascular information will serve as a guide to help clinicians recognize and treat other similar, but reversible through surgery, frontal network syndromes.

## 3. Discussion

This case seeks to underscore how a giant non-dominant sphenoid wing meningioma can silently construct a highly structured dysexecutive–apathetic syndrome that is clinically almost indistinguishable from a primary neurodegenerative disease, yet remains substantially reversible when approached as a network lesion and decompressed with a skull-base, venous-sparing strategy. We aimed to use the density of clinical, imaging, and surgical detail not to exaggerate the singularity of this case, but to show how an apparently “degenerative” frontal syndrome may conceal a surgically correctable skull-base pathology.

### 3.1. Pseudo-Degenerative Presentation at the Limit of the Meningioma Spectrum

Cohort data indicate that many patients with intracranial meningiomas display preoperative deficits in attention, executive function, and working memory, particularly when tumors are large and frontal. Yet, in routine practice, these deficits are often under-characterized, and many reports rely on global screening tools alone [14]. Our patient lies at the extreme of this spectrum: functionally, she presented with near-complete loss of instrumental autonomy and a severe dysexecutive–apathetic syndrome; psychometrically, she exhibited a tightly documented frontal phenotype across multiple domains (MoCA, FAB, Boston Naming Test, Token Test, NPI, IADL, Barthel, Tinetti, Clinical Frailty Scale), while language and basic comprehension were relatively preserved.

We consider this level of quantification to be one of the distinctive aspects of the case. It allowed us to show that a non-dominant sphenoid wing lesion, classically associated with visual or cranial nerve presentations, can instead reproduce a bvFTD-like syndrome with late seizure and pyramidal signs, in a manner that is not merely anecdotal but supported by standardized scores. We intend that this detailed profile may help clinicians recognize similar “pseudo-bvFTD” constellations in patients who might otherwise be managed primarily in psychiatric or dementia pathways.

### 3.2. Network-Level Interpretation and Staged Reversibility

Another particularity of this case is the explicit integration of network thinking into the clinical reasoning. The preoperative MRI did not simply demonstrate “a large right sphenoid wing mass with edema”; it showed vasogenic edema propagating from the frontal base through the centrum semiovale and corona radiata into the anterior limb of the internal capsule, anatomically aligned with fronto-striato-thalamo-cortical and corticospinal tracts. This anatomy corresponds closely to the patient’s combination of apathy, dysexecutive syndrome, dynamic aphasia, left pyramidal signs, and frontal gait disturbance. Postoperatively, the improvement was not only global but patterned: early normalization of vigilance and initiative, gradual restoration of executive control, and progressive recovery of gait and functional independence. Serial scores (NIHSS, GCS, FAB, MoCA, NPI, Barthel, mRS, Tinetti, Karnofsky, CFS) documented a stepwise reversal of frontal network failure rather than a vague “clinical improvement”. We aimed to show that this trajectory is coherent with a network model of meningioma-related cognitive dysfunction, in which decompression and edema regression allow partial restoration of disrupted circuits, especially when venous and white-matter integrity are preserved.

By presenting the case in this way, we intend to complement existing series that report group-level trends with an individual-level narrative where behavior, imaging, and operative strategy can be directly aligned.

### 3.3. Skull-Base, Venous-Sparing Strategy Tailored to a “Cognitive” Meningioma

Maximal Safe Resection as First-Line Strategy. The rationale for choosing maximal safe resection as the first line of treatment in this patient’s case was based on several factors. The patient presented with a rapidly deteriorating pseudo-bvFTD syndrome that was objectively worsening her independence and included a new-onset seizure, evolving pyramidal weakness, decreased level of consciousness, and a severely displaced midline brain structure. These findings were consistent with a severely compromised neurological status. The preoperative MRI studies had demonstrated a highly favorable anatomical configuration to allow maximal safe resection (the arteries were displaced rather than encircled by the tumor, and there was no compromise to the venous system). Therefore, the goal of maximal safe resection was to provide the most complete possible decompression of the tumor and thereby preserve as much of the patient’s neural networks as possible while minimizing the long-term need for additional treatments such as radiation therapy.

Alternative Strategies. There are many different approaches to managing large sphenoid wing meningiomas. For example, the surgeon may choose to perform staged surgeries if the tumor is excessively vascular or if prolonged operative time could result in significant post-operative morbidity. Alternatively, the surgeon may elect to remove as much of the tumor as possible and then treat the remaining tumor with fractionated radiotherapy or stereotactic radiosurgery to avoid the risks associated with surgical removal of the tumor at the base of the skull. Finally, some surgeons will adopt a more conservative approach and only partially resect the tumor if the patient’s condition does not warrant a more aggressive resection. This decision-making process involves consideration of both the patient’s medical history and current health status, as well as the rate of growth of the tumor and the severity of the patient’s symptoms.

Sphenoid wing meningiomas are well known for their technical challenges, particularly when large, hyperostotic, or medial. Many series emphasize visual, cranial nerve, and vessel-related risks; relatively few describe in detail how venous anatomy and frontal networks are taken into account when planning surgery for patients whose dominant complaint is cognitive or behavioral [15].

In our case, preoperative MR angiographic and venographic imaging demonstrated a giant right fronto-temporo-basal mass displacing, but not occluding, cortical veins and preserving parasagittal drainage. This information did more than simply reassure us about sinus patency; it allowed us to design a pterional, skull-base-flattening approach with explicit venous-sparing priorities, including the following:

- Extensive drilling of the lesser sphenoid wing to shorten the basal working distance and limit frontal retraction;
- Early basal devascularisation to decrease intratumoral pressure before any traction on edematous frontal cortex;
- Staged internal debulking to transform the tumor into a collapsible shell before manipulating Sylvian vessels or medial structures;
- Arachnoid-preserving dissection around the Sylvian fissure and convexity;
- Deliberately conservative handling of medial venous adhesions, with acceptance of leaving thin capsular remnants on critical veins.

Taken together, these steps were not unique in isolation, yet in this context they were explicitly chosen to protect both venous physiology and the very networks whose failure defined the patient’s presentation. We do not claim a new operative technique; rather, we aimed to show how existing skull-base principles can be deliberately applied to a case primarily defined by cognitive collapse, with subsequent functional recovery as an indirect validation of this approach. To contextualize this case, we synthesized key important studies (Table 1) on meningioma-related cognitive dysfunction, skull-base surgery, network disruption, and tumor-bvFTD mimics.

### 3.4. Histopathological, Biological, and Network Effects

The histologic appearance of the tumor was consistent with that of a typical meningioma; it had no characteristics that indicated an aggressive growth pattern. The disparity between benign histologic findings and catastrophic functional deficits in this patient serves as a valuable illustration of how, in large skull base tumors, the extent of clinical impairment may result from the combined effects of tumor duration, tumor volume, and the compromise of veins and white matter, rather than merely the growth rate of the tumor.

Increasingly, molecular biomarkers, including TERT promoter mutations, loss of CDKN2A/B, and DNA methylation patterns, are being used in current classifications to better define risk in patients undergoing treatment for meningiomas. These tools provide improved predictions regarding tumor recurrence and tumor aggressiveness, especially in those tumors that are higher grade or have atypical histologic findings [26]. Since the major issue in this case was network collapse rather than early recurrence, the most important lesson derived from this case is complementary: Even benign, low-grade sphenoid wing meningiomas can cause severe, though potentially reversible, neurocognitive syndromes if allowed to grow unabated adjacent to the frontal lobes and their associated tracts. We hope to reinforce the notion that “psychiatric” or “degenerative” pathways for many years do not preclude a structurally treatable tumor, even when cytologic analysis reveals the tumor to be low grade.

### 3.5. Radiation and Systemic Treatments

Skull-base meningiomas are currently managed using a combination of surgical intervention, radiation therapy, and systemic therapy in selected cases of recurrent tumors. Radiation therapy using conformal, fractionated irradiation and stereotactic radiosurgery can provide excellent local control for small residual skull-base tumors. However, the delivery of radiation therapy to the optic nerves and proximal vasculature can limit its utility [27]. Similarly, systemic therapies (e.g., antiangiogenic agents, multitargeted kinase inhibitors, and somatostatin receptor-targeted approaches) can stabilize disease in selected patients with progressive or higher-grade meningiomas. However, responses to systemic therapies have been inconsistent and based on data collected from relatively small, diverse populations [28].

As illustrated by this patient’s clinical course, in select cases of skull-base meningiomas, maximal safe resection may be sufficient to alleviate mass effect, restore network function, and leave no measurable residual tumor. Thus, we do not consider adjuvant radiation therapy or systemic therapy to be necessary in all cases; however, we also recognize that these modalities play a crucial role in the management of skull-base meningiomas where surgical resection is limited by vascular or neural involvement, or where molecular characteristics indicate early recurrence. Therefore, the present case represents one of the few cases where skull-base surgery alone has transformed a “pseudo-degenerative” state to a nearly independent life.

### 3.6. Clinical Lessons and Future Directions

Recognizing that the present report represents a single case, and thus does not account for the diversity of presentations of sphenoid wing meningiomas, we wish to highlight a number of practical lessons learned from this case:

Frontal dementia with unilateral motor weakness and seizures must be evaluated to rule out a structural lesion (e.g., frontal or skull-base tumor). A slowly progressive dysexecutive–apathetic syndrome with later onset seizure and unilateral pyramidal sign(s), regardless of age, should prompt evaluation with contrast-enhanced MRI to evaluate the possibility of a frontal or skull-base tumor [29].

Preoperative knowledge of the major networks and venous drainage of a sphenoid wing meningioma can assist surgeons in selecting an appropriate approach, determining the optimal order of dissection and debulking, and determining whether to sacrifice portions of critical veins [20].

Systematic assessment of cognitive and behavioral functions, in addition to global evaluations and imaging studies, provides value in documenting that some syndromes appear to represent primary degenerative processes, but are actually structurally reversible, and quantifying the benefits of targeted decompression [30].

Through this detailed presentation of a single case, we have attempted to place large, non-dominant sphenoid wing meningiomas as models of surgically correctable failures of the frontal network. We expect that this case will prompt clinicians to maintain a structural diagnosis of consideration in atypical “dementia” presentations, and to think about both circuits and veins (not just size and location of the tumor) when developing a plan for the surgical management of skull-base meningiomas.

## 4. Conclusions

In this report, we have attempted to transform a large, non-dominant sphenoid wing meningioma from a “mass occupying space” to a potentially reversible disorder of the frontal networks. While traditionally, we would evaluate meningiomas based on their size and histological characteristics alone, our objective was to demonstrate how behavioral, cognitive, and functional assessments could provide a comprehensive profile that was indistinguishable from primary neurodegenerative disorders encountered clinically, yet completely compatible with a surgically correctible substrate.

Practically speaking, we believe that this case supports three points. The first point is that slowly progressive frontal syndromes with late onset seizure activity and asymmetry are not always dementia and should be evaluated for structural pathologies with high-quality, contrast-enhanced imaging before being labeled as “pure” dementia. The second point is that the relevant anatomy when evaluating skull base meningiomas includes not just blood vessels, cranial nerves and bony structures, but also venous drainage pathways and white matter pathways that support motivational behavior, executive function and gait. The third point is that a quantitative evaluation of recovery using formal, structured follow-up assessments is much more valuable than subjective descriptions when assessing outcomes in these types of patients, since significant and staged recoveries of networks can occur even in cases where there is significant clinical deterioration.

This case represents a beginning point rather than an end point; we hope that this study will facilitate the utilization of formal, standardized cognitive testing and network-based imaging techniques in meningioma patients who are initially diagnosed with psychiatric or degenerative labels. Additionally, we hope that future studies will investigate the integration of imaging modalities (connectomics), molecular markers, and long-term functional trajectories. In a simple and direct manner, we want to remind physicians that even though a patient appears to have severe frontal dysfunction, a treatable skull base lesion may still exist, and if properly planned, surgical interventions can result in restoring much more than radiologic normalcy.

## Figures and Tables

**Table 1:** Key literature contextualizing frontal cognitive phenotypes, network-level mechanisms, and skull-base strategy relevant to the present case.

References	Study Type/Cohort	Key Finding (High-Yield)	Relevance to This Case
[16]	Prospective neurocognitive cohort (supratentorial meningiomas; frontal vs. non-frontal)	Frontal/large tumors disproportionately impair executive/attention domains; partial reversibility after resection.	Supports interpreting our quantified dysexecutive–apathetic phenotype as tumor-related and potentially reversible when decompression is achieved.
[17]	Longitudinal serial testing after meningioma surgery	Global cognition often improves, yet executive deficits may persist.	Frames our unusually deep, measurable frontal recovery as notable and highlights the potential value of optimized decompression/venous preservation.
[18]	Location–laterality cohort (frontal/temporal/parietal/skull base; left vs. right)	Right frontal/right skull-base lesions show characteristic dysexecutive–behavioral profiles.	Aligns with our right non-dominant sphenoid wing lesion producing a pseudo-bvFTD presentation with relatively preserved core language.
[19]	Skull-base surgical series (lateral/medial sphenoid wing meningiomas)	Pterional/extended pterional approaches yield high (near-)total resection with definable cranial nerve/vascular risks; seizure/visual outcomes often improve.	Establishes standard skull-base safety/efficacy; our case applies the same strategy to a primarily cognitive–behavioral indication.
[20]	Medial/clinoidal sphenoid wing cohort (optic apparatus and ICA/MCA relationships)	Outcomes and complications track degree of encasement; decompression is outcome-critical.	Places our non-encasing globoid tumor at the lower vascular-risk end, supporting Simpson-oriented, function-preserving resection.
[21]	Tumor connectome studies (rs-fMRI/diffusion; including meningiomas)	Tumors and edema disrupt connectivity beyond the lesion core (DMN/salience/frontoparietal control networks).	Provides mechanistic context for our structural “network-relevant” interpretation (not patient-specific functional imaging).
[22]	Anterior skull-base rs-fMRI study (frontal base meningiomas)	Frontal connectivity abnormalities partially normalize postoperatively and may parallel clinical improvement.	Conceptually parallels our staged improvements in frontal testing/apathy after decompression and edema regression.
[23]	Tumor–bvFTD mimic reports	FTD-like syndromes may improve after mass lesion treatment; delayed imaging is common.	Anchors the pseudo-bvFTD diagnostic pitfall; our case adds unusually dense longitudinal metrics and a surgically remediable sphenoid wing etiology.
[24]	Molecular prognostication cohorts (integrated markers across grades)	Molecular markers stratify recurrence/survival; biologic grade ≠ functional impact.	Reinforces that “benign” histology can still drive profound network-level disability that is reversible when mass effect is relieved.
[25]	Systemic therapy series (progressive/unresectable meningioma)	Systemic agents mainly stabilize disease; functional recovery is less consistent.	Contrasts with our case: complete anatomical skull-base resection alone produced major cognitive/functional restoration.

## Data Availability

The data presented in this study are available upon request from the corresponding author; however, they are not publicly available due to privacy and ethical restrictions related to patient confidentiality.
